# C-peptide as a Therapy for Kidney Disease: A Systematic Review and Meta-Analysis

**DOI:** 10.1371/journal.pone.0127439

**Published:** 2015-05-20

**Authors:** James A. Shaw, Partha Shetty, Kevin D. Burns, Dean Fergusson, Greg A. Knoll

**Affiliations:** 1 Department of Medicine, University of Ottawa, Ottawa, Ontario, Canada; 2 Division of Nephrology, Kidney Research Centre, Ottawa Hospital Research Institute, Ottawa, Ontario, Canada; 3 Clinical Epidemiology Program, Ottawa Hospital Research Institute, Ottawa, Ontario, Canada; University of São Paulo Medical School, BRAZIL

## Abstract

C-peptide has intrinsic biological activity and may be renoprotective. We conducted a systematic review to determine whether C-peptide had a beneficial effect on renal outcomes. MEDLINE, EMBASE, and the Cochrane Central Databases were searched for human and animal studies in which C-peptide was administered and renal endpoints were subsequently measured. We identified 4 human trials involving 74 patients as well as 18 animal studies involving 35 separate experiments with a total of 641 animals. In humans, the renal effects of exogenously delivered C-peptide were only studied in type 1 diabetics with either normal renal function or incipient nephropathy. Pooled analysis showed no difference in GFR (mean difference, -1.36 mL/min/1.73 m2, p = 0.72) in patients receiving C-peptide compared to a control group, but two studies reported a reduction in glomerular hyperfiltration (p<0.05). Reduction in albuminuria was also reported in the C-peptide group (p<0.05). In diabetic rodent models, C-peptide led to a reduction in GFR (mean difference, -0.62 mL/min, p<0.00001) reflecting a partial reduction in glomerular hyperfiltration. C-peptide also reduced proteinuria (mean difference, -186.25 mg/day, p = 0.05), glomerular volume (p<0.00001), and mesangial matrix area (p<0.00001) in diabetic animals without affecting blood pressure or plasma glucose. Most studies were relatively short-term in duration, ranging from 1 hour to 3 months. Human studies of sufficient sample size and duration are needed to determine if the beneficial effects of C-peptide seen in animal models translate into improved long-term clinical outcomes for patients with chronic kidney disease. (PROSPERO CRD42014007472)

## Introduction

Between 2007 and 2009 the prevalence of chronic kidney disease (CKD) in Canada was 12.5% and is expected to rise in the coming years due to high rates of risk factors such as diabetes and hypertension [1]. CKD and end stage renal disease (ESRD) are associated with increased morbidity and mortality as well as increased health care costs [2]. Thus, new disease modifying therapies are needed to slow or stop the progression of CKD to end stage.

C-peptide, a protein released during insulin secretion, was previously thought to be inert, but has now been recognized as a physiologically active molecule with numerous potential cellular targets [3,4]. Studies involving type 1 diabetics have suggested improved renal function following C-peptide administration [5,6].

Further support for a renoprotective role for C-peptide comes from observational studies in pancreas transplant recipients suggesting that the improved renal function post-transplant may be mediated, in part, by repletion of C-peptide [7,8]. Others have suggested that higher serum C-peptide concentration in diabetics is correlated with reduced risk of microvascular complications (reviewed in [9]). However, it remains unclear whether C-peptide could provide objective benefit to patients with kidney disease. The aim of this systematic review was to determine the effect of exogenously delivered C-peptide on renal relevant outcomes in humans and other mammalian species.

## Materials and Methods

A detailed protocol for this review has been published [10]. In brief, MEDLINE (1946 to January 17, 2014), EMBASE (1947 to January 17, 2014), and the Cochrane Central Databases (1991 to January 17, 2014) were searched using keywords related to C-peptide and kidney disease. The search strategy was intentionally broad to be as sensitive as possible. Titles and abstracts of search results were screened by two independent reviewers (JAS and PS) for potential inclusion, and differences were reconciled by a third party. Full text of screened papers were independently reviewed by the same two investigators and selected for inclusion based on the following criteria:

1. The experimental subjects were either humans or other mammals of any age;
2. The study intervention involved the administration of exogenous C-peptide to subjects;
3. The reported outcomes were relevant markers of kidney function, kidney disease, requirement for renal replacement therapy, or mortality.

*In vivo*, *ex vivo*, and *in vitro* studies with only cellular or molecular endpoints were excluded. Case reports, narrative reviews, and non-English publications were also excluded.

Data extraction was facilitated by a standardized form used by each reviewer. The following were abstracted from each study: details on study design, subject characteristics, C-peptide type and dose, and outcomes [glomerular filtration rate (GFR), serum creatinine, proteinuria, albuminuria, hematuria, blood pressure, renal blood flow, urine electrolytes, kidney size, kidney histology, and requirements of renal replacement therapy]. When studies reported the same outcome in different units, all data was converted to the same units mathematically. When data was only available in figures, the GNU image manipulation program (GIMP 2.8; http://www.gimp.org) was used to extract data. Methodological quality was assessed with the Cochrane Collaboration’s tool for assessing risk of bias [11].

For continuous variables, mean differences between control and C-peptide groups were calculated and pooled, when appropriate, using a random effects model. If all data points were not reported in crossover trials, only the first period was included [12]. When multiple doses were given to the same experimental subject without sufficient wash-out time, only the first dose was considered. The I^2^ statistic was used as a measure of heterogeneity; it describes the percentage of variability due to heterogeneity rather than chance alone [12]. Review Manager (RevMan 5.2.8; http://tech.cochrane.org/revman) software was used for statistical calculation and forest plot creation. Data was considered significant at p<0.05.

## Results

### Study Characteristics and Disease Models

**Fig 1:**
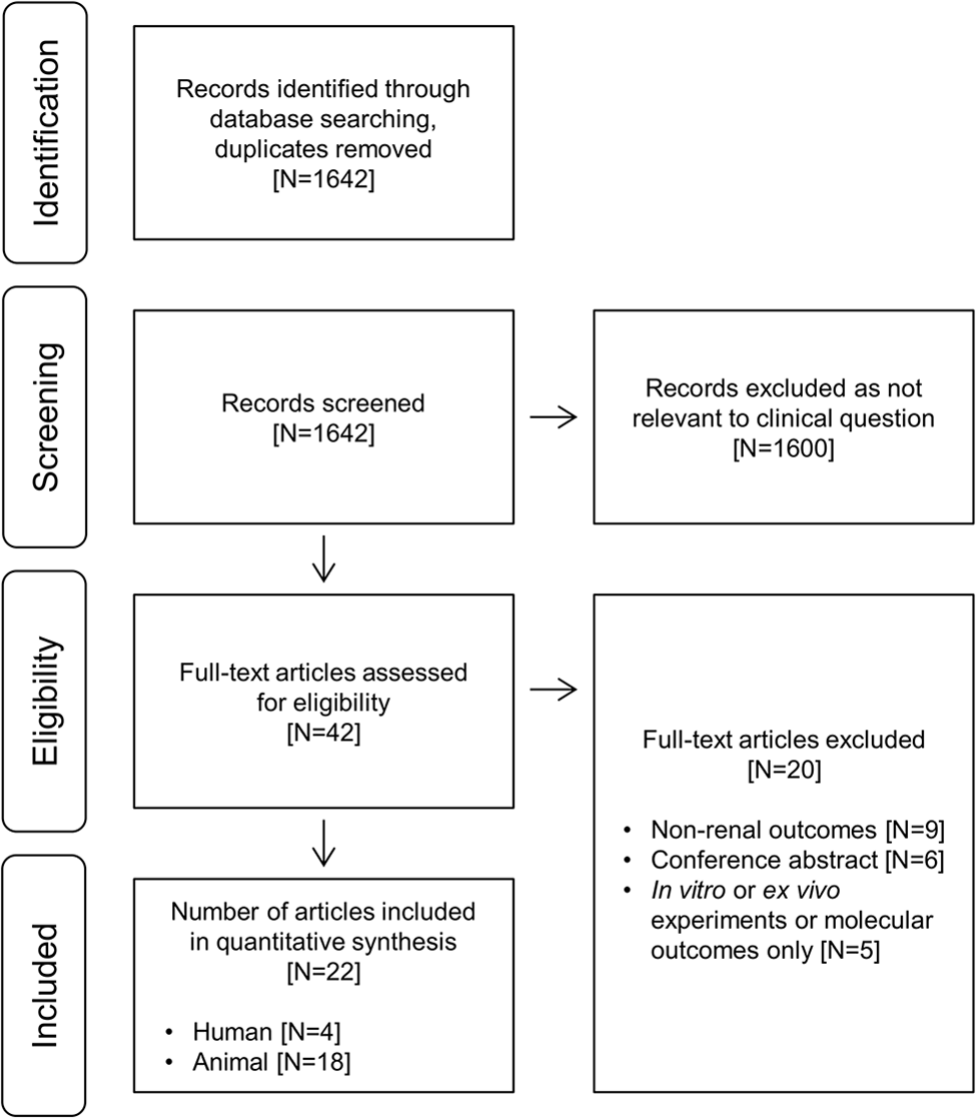
PRISMA (Preferred Reporting Items for Systematic Reviews and Meta-Analyses) flow diagram of the systematic literature search.

Our initial search identified 1642 records. Most were excluded at this stage as they were not relevant to the study question. Forty-two articles were reviewed in full-text and 22 met inclusion criteria. There were four human studies involving a total of 74 patients, and 18 animal publications which together described 35 separate experiments involving a total of 641 animals (Fig 1). Rationale for the exclusion of each full-text article is available in the supporting information (S1 File).

All human studies were conducted in type 1 diabetics with either normal GFR or hyperfiltration, with or without evidence of microalbuminuria. The age of participants ranged from 18–40 years old and the percentage of male participants ranged from 50–81%. All human studies were prospective; two were randomized double-blind trials [5,6], and two compared groups without explicitly stating the study design [13,14]. Human C-peptide was administered by intravenous or subcutaneous infusion for either 1 hour or 4 weeks, or subcutaneously three times per day for 3 months. All control patients were given saline without C-peptide. Human studies are summarized in Table 1. Allocation concealment was not well reported in the human studies but otherwise risk of bias was low (Table 2).

**Table 1:** Study characteristics of human studies.

Reference	Year	Country	N	Disease Studied	C-Peptide Dose	C-Peptide Route & Duration	Outcomes of Interest
**Johansson *et al*. [5]**	2000	Sweden	21	DM1	225 nmol QAM + QHS, plus 150 nmol Qsupper	Subcutaneous 3 months	GFR, UAE, UAC, HbA1c, Glucose
**Johansson *et al*. [6]**	1993	Sweden	18	DM1	Equimolar to insulin infusion dose	Subcutaneous Infusion4 weeks	GFR, FF, RPF, UAE, HbA1c, Glucose, BP
**Johansson *et al*. [13]**	1992	Sweden	21	DM1	25 pmol/kg/min x 1.5 min, then 10 pmol/kg/min x 6.5 min, then 5 pmol/kg/min x 52 min	Intravenous Infusion 1 hour total	GFR, RPF, FF, Glucose
**Sjöberg *et al*. [14]**	1991	Sweden	14	DM1	25 pmol/kg/min x 1.5 min, then 10 pmol/kg/min x 6.5 min, then 5 pmol/kg/min x 52 min	Intravenous Infusion 1 hour total	RPF, Glucose

DM1 = Diabetes Mellitus Type 1; GFR = glomerular filtration rate; RPF = Renal Plasma Flow; FF = filtration fraction; UAE = urine albumin excretion; UAC = urine albumin:creatinine ratio; HbA1c = Hemoglobin A1c; BP = Blood Pressure.

**Table 2:** Risk of bias in human studies.

Reference	Adequate sequence generation?	Allocation Concealment?	Blinding?	Incomplete Outcome Data Addressed?	Free of Selective Reporting?	Free of other sources of bias?
**Johansson *et al*. [5]**	Yes	Unclear	Yes	Yes	Yes	Yes
**Johansson *et al*. [6]**	Yes	Unclear	Yes	Yes	Yes	Yes
**Johansson *et al*. [13]**	Unclear	Unclear	Unclear	Yes	Yes	Yes
**Sjöberg *et al*. [14]**	Unclear	Unclear	Unclear	Yes	Yes	Yes

Yes = low risk of bias

Of the 18 animal publications, 15 examined the effect of C-peptide using a model of diabetic nephropathy, one studied non-diabetic CKD [15], and two examined its effects on acute kidney injury (AKI) in either a model of hemorrhagic shock in Wistar rats [16] or endotoxin-mediated shock in Swiss albino mice [17]. Of the 15 diabetic nephropathy publications, 14 injected streptozotocin into either Sprague-Dawley rats (11 publications), Wistar rats (2 publications), or C57/B16J mice (1 publication) to model type 1 diabetes, and the remaining study used Zucker fatty diabetic rats to model type 2 diabetes. Following injection with streptozotocin there was typically a variable period of monitoring time from 24 hours to 8 weeks, with or without concurrent insulin supplementation, until treatment with C-peptide or vehicle was initiated. For animal studies, rat or human C-peptide was administered most commonly at 50 pmol/kg/min either subcutaneously or intravenously. The duration of drug exposure varied from less than one hour to 3 months. Only two animal studies used a scrambled amino acid peptide as a control [18,19], and this proved to be no different than vehicle control [20], which was commonly used. Non-diabetic studies were carried out in Dahl salt-sensitive (SS/jr) rats or wild-type Sprague-Dawley or Wistar rats. All animal studies used only male animals, but the rationale and implications for this were not explicitly addressed in any study. Most publications described more than one unique experiment, and each experiment typically involved between 5–10 animals per group to ensure reproducibility and consistency of results. Animal studies are summarized in Table 3.

**Table 3:** Study design characteristics of animal studies.

Reference	Year	Country	N	Disease Model	Species	C-peptide Dose	Outcomes of Interest
**Flynn *et al*. [21]**	2013	USA	43	STZ	Sprague-Dawley Rat	50 pmol/kg/min SC infusion x 2–4 weeks	GFR, RPF, RVR, KWBW, UAE*, UPE*, GV*, HbA1c, Glc*, MAP
**Nakamoto *et al*. [22]**	2013	Japan	32	STZ	Wistar Rat	50 pmol/kg/min IV infusion x 4–5 hours	Relative Sieving Coefficient, HbA1c
**Sawyer *et al*. [15]**	2012	USA	21	Non-diabetic CKD induced by high salt diet	Dahl salt-sensitive (SS/jr) Rat	50 pmol/kg/min SC infusion x 4 weeks	GFR, KWBW, UAE*, UPE*, GSI*, TIFI*, Glc, MAP
**Yang *et al*. [23]**	2011	China	24	DM2 genetic model	Zucker diabetic fatty Rat	250 or 500 nmol/kg SC daily x 12 weeks	KWBW, GV, GMBT, ECM
**Chima *et al*. [16]**	2011	USA	15	Shock	Wistar Rat	280 nmol/kg IV infusion x 3 hours	Serum Creatinine, MAP
**Sun *et al*. [24]**	2010	China	36	STZ	Sprague-Dawley Rat	130 nmol/kg SC Q12H x 8 weeks	KWBW, ECM, GV
**Stridh *et al*. [25]**	2009	Sweden	10	STZ	Sprague-Dawley Rat	0.2 fmol Q2weeks x 2 doses	GFR
**Nordquist *et al*. [20]**	2009	Sweden	36	STZ	Sprague-Dawley Rat	50 pmol/kg/min IV x 40 minutes	GFR*, FF*, RVR, RPF, MAP
**Kamikawa *et al*. [26]**	2008	Japan	48	STZ	Sprague-Dawley Rat	35 pmol/kg/min SC infusion x 1 week	GV*, Glc*
**Nordquist *et al*. [27]**	2007	Sweden	45	STZ	Sprague-Dawley Rat	50 pmol/kg/min SC infusion x 1 week	GFR*, UNa, UK, Glc, MAP
**Vish *et al*. [17]**	2007	USA	66	Shock	Male Swiss albino mice	70 or 140 nmol/kg IP x 2 doses 3 hours apart	Mortality
**Maezawa *et al*. [18]**	2006	China	28	STZ	C57/B16L mice	290 pmol/kg/min SC infusion x 24 hours	CrCl, UAE, Glc, MAP
**Rebsomen *et al*. [28]**	2006	France	24	STZ	Sprague-Dawley Rat	50 pmol/kg/day IP infusion x 28 days	CrCl, UPE, UNa, Glc
**Samnegard *et al*. [29]**	2005	Sweden	47	STZ	Wistar Rat	50 pmol/kg/min SC infusion x 4 weeks	GFR, UAE, UNa, UK, GBMT, ECM, GV, Glc
**Samnegard *et al*. [30]**	2004	Sweden	42	STZ	Sprague-Dawley Rat	50 pmol/kg/min IV infusion x 60 minutes	GFR*, RPF*, FF*, UNa, UK, Glc, MAP*
**Huang *et al*. [31]**	2002	Germany	71	STZ	Sprague-Dawley Rat	3 to 300 nmol/kg/hour IV infusion x 30 minutes	GFR*, RPF*, RVR*, UNa*, UK*, UAE, UPE*, Glc, MAP
**Samnegard *et al*. [32]**	2001	Sweden	21	STZ	Sprague-Dawley Rat	50 pmol/kg/min IV infusion x 2 weeks	GFR, UNa, GV UK, UAE*, RFR, Glc
**Sjöquist *et al*. [19]**	1998	Sweden	32	STZ	Sprague-Dawley Rat	0.5 nmol/min/kg IV infusion x 140 minutes	GFR*, UNa*, UK*, UPE, Glc, MAP

STZ = streptozotocin-induced diabetes (type 1 diabetes); DM2 = type 2 diabetes; SC = subcutaneous; IV = intravenous; IP = intraperitoneal; GFR = glomerular filtration rate; CrCl = 24 hour creatinine clearance; RPF = renal plasma flow; RVR = renal vascular resistance; FF = filtration fraction; KWBW = Kidney weight:body weight ratio; UAE = urine albumin excretion; UPE = urine protein excretion; GV = glomerular volume; GBMT = glomerular basement membrane thickness; ECM = Extracellular matrix area fraction of glomerular cross section; UNa = urinary sodium excretion; UK = urinary potassium excretion; RFR = Renal functional reserve, GSI = Index of glomerulosclerosis, TIFI = Index of tubulointerstitial fibrosis, HbA1c = hemoglobin A1c, Glc = plasma glucose, MAP = mean arterial pressure,

* = estimated data extracted electronically from study Figure (see Methods). N reflects the total number of animals in the study overall, not for a particular endpoint.

Formal risk of bias assessment was attempted for animal studies as per our analysis protocol but due to different reporting standards and conventions compared to human clinical trials there was often no description of randomization, allocation concealment, or blinding (S1 Table). We did not identify any publications with evidence of incomplete data, selective reporting, or other source of bias in any of the animal studies included in our analysis.

### C-peptide in Humans

**Fig 2:**
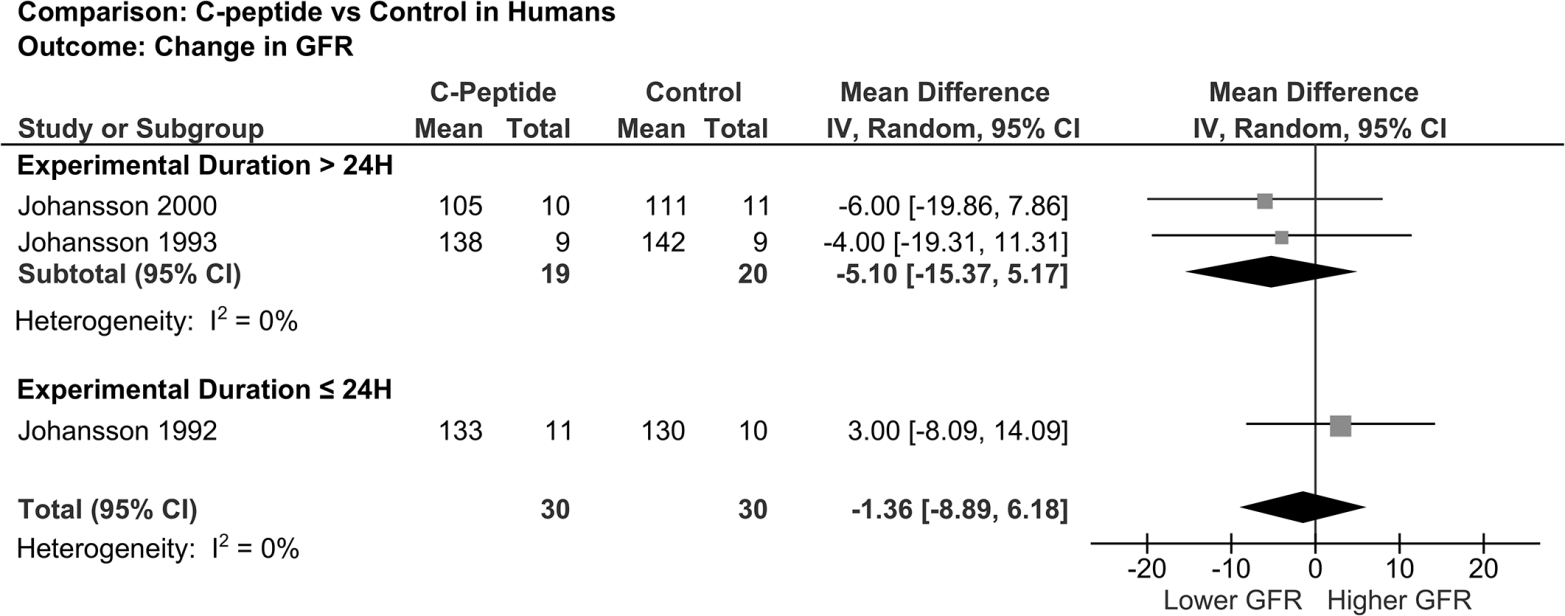
Forest plot showing pooled mean difference in GFR in human studies. The box size reflects relative weight.

The effect of C-peptide on GFR in patients with type 1 diabetes, as reported in three studies, was not significantly different from control regardless of study duration when comparing post-intervention values (overall pooled mean difference -1.36 mL/min/1.73 m^2^; 95% CI -8.89 to 6.18) (Fig 2). However, two studies reported a reduction in glomerular hyperfiltration with C-peptide by comparing the change from baseline (p<0.05)[6,13]. Renal plasma flow was examined in three studies and there was no significant difference between patients receiving C-peptide compared to control (pooled mean difference 32.53 mL/min/1.73 m^2^; 95% CI -25.27 to 90.33) (Table 4).

**Table 4:** Outcome summary of pooled mean differences in human studies.

Outcome	Duration	No. of Studies [ref.]	N	Mean Difference (95% CI)	Units	P	I^2^
**Renal Plasma Flow**	>24 h	1^[^ ^6^ ^]^	18	52.0 (-51.3, 155.3)	mL/min/1.73m^2^	0.32	
	≤24 h	2^[^ ^13^ ^,^ ^14^ ^]^	45	23.7 (-46.1, 93.4)	mL/min/1.73m^2^	0.51	0%
	All	3	63	32.5 (-25.3, 90.3)	mL/min/1.73m^2^	0.27	0%
**Plasma Glucose**	>24 h	2^[^ ^5^ ^,^ ^6^ ^]^	39	-2.0 (-7.7, 3.7)	mmol/L	0.49	79%
	≤24 h	1^[^ ^13^ ^]^	21	0.0 (-0.3, 0.3)	mmol/L	1.00	-
	All	3	60	-1.0 (-3.9, 1.8)	mmol/L	0.47	71%
**HbA1c**	>24 h	2^[^ ^5^ ^,^ ^6^ ^]^	39	0.1 (-0.7, 0.8)	%	0.90	0%

Positive mean difference indicates higher numbers in the C-peptide group. HbA1c = hemoglobin A1c.

Two studies reported urine albumin excretion rates, but only one included a measurement of variability, so the data could not be pooled. Johansson *et al*. [6] showed that the end-of-study urine albumin excretion was significantly lower in the C-peptide group compared to control (p<0.05). In a separate study Johansson *et al*. [5] reported that C-peptide reduced urine albumin excretion (p<0.01) and also reduced urine albumin/creatinine ratio by approximately 30% after 3 months (p<0.01), whereas control subjects did not have a significant change in albuminuria during the study period.

C-peptide had no significant effect on plasma glucose or hemoglobin A1c (Table 4). No human studies reported need for renal replacement therapy, renal histology, hematuria, cardiovascular events, or mortality as endpoints. Blood pressure data were only reported in one study [6], and informally commented on in another [5], and in both studies was unaffected by C-peptide.

### C-peptide in Diabetic Animals

**Fig 3:**
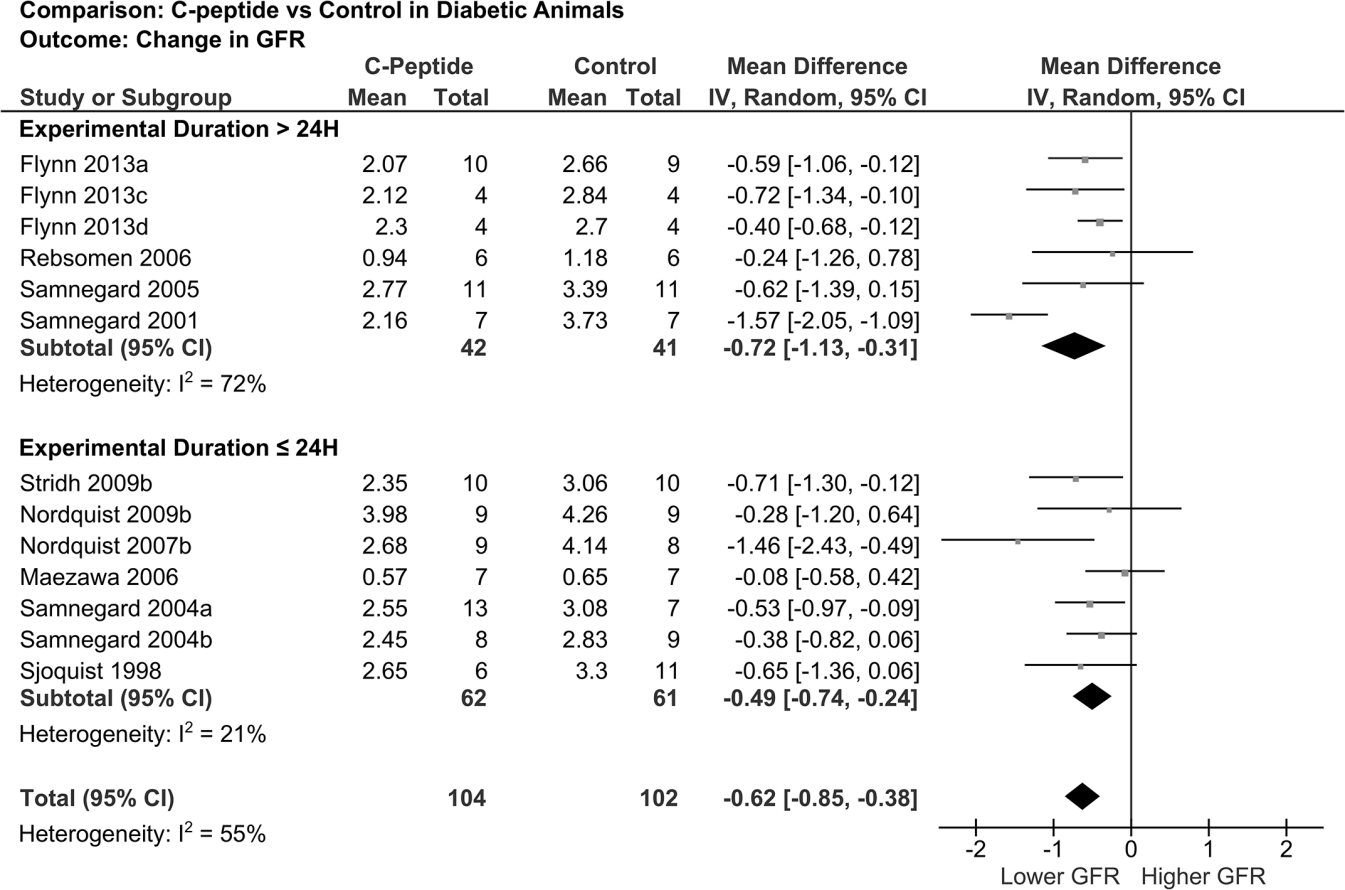
Forest plot showing pooled mean difference in GFR in diabetic animal studies. Flynn 2013a,c = low insulin dose; Flynn 2013d = high insulin dose. Samnegard 2004a = absence of captopril, Samnegard 2004b = presence of captopril. The box size reflects relative weight.

There were 10 publications with 13 unique experiments that reported GFR in diabetic animals. Overall, C-peptide induced a statistically significant reduction in GFR, thereby reducing diabetes-induced glomerular hyperfiltration (pooled mean difference -0.62 mL/min; 95% CI -0.85 to -0.38) (Fig 3). This finding was consistent for experiments lasting >24 hours (pooled mean difference -0.72 mL/min; 95% CI -1.13 to -0.31) as well as for those ≤24 hours (pooled mean difference -0.49 mL/min; 95% CI -0.74 to -0.24) (Fig 3). Nordquist *et al*. [20] and Samnegard *et al*. [30] reported reduction in filtration fraction in diabetic animals with C-peptide compared to control. C-peptide did not affect renal plasma flow or renal vascular resistance (Table 5).

**Table 5:** Outcome summary of pooled mean differences in diabetic animal studies.

Outcome	Duration	Unique Exp. [ref.]	N	Mean Difference (95% CI)	Units	P	I^2^
**Renal Plasma Flow**	>24 h	1^[^ ^21^ ^]^	19	-0.3 (-3.2, 2.6)	mL/min	0.84	
	≤24 h	1^[^ ^20^ ^]^	18	0.0 (-2.8, 2.8)	mL/min	1.00	
	All	2	37	-0.1 (-2.2, 1.9)	mL/min	0.89	0%
**Renal Vascular Resistance**	>24 h	1^[^ ^21^ ^]^	19	0.8 (-2.9, 4.5)	mmHg/mL/min	0.68	
	≤24 h	1^[^ ^20^ ^]^	18	-0.6 (-4.1, 2.9)	mmHg/mL/min	0.74	
	All	2	37	0.1 (-2.5, 2.6)	mmHg/mL/min	0.97	0%
**Urine Albumin Excretion**	>24 h	3^[^ ^21^ ^,^ ^29^ ^,^ ^32^ ^]^	55	-0.3 (-1.2, 0.6)	mg/day	0.56	75%
	≤24 h	1^[^ ^18^ ^]^	14	-0.2 (-0.3, -0.1)	mg/day	0.001	
	All	4	69	-0.2 (-0.6, 0.2)	mg/day	0.36	62%
**Urine Protein Excretion**	>24 h	2^[^ ^21^ ^,^ ^28^ ^]^	31	-332.5 (-954.8, 289.8)	mg/day	0.30	97%
	≤24 h	1^[^ ^19^ ^]^	17	-14.3 (-22.7, -5.9)	mg/day	0.0009	
	All	3	48	-186.3 (-372.0, -0.5)	mg/day	0.05	95%
**Urine Na Excretion**	>24 h	3^[^ ^28^ ^,^ ^29^ ^,^ ^32^ ^]^	48	-0.1 (-0.4, 0.3)	μmol/min	0.71	0%
	≤24 h	4^[^ ^19^ ^,^ ^27^ ^,^ ^30^ ^]^	71	0.3 (0.1, 0.6)	μmol/min	0.004	0%
	All	7	119	0.2 (0.0, 0.4)	μmol/min	0.05	7%
**Urine K Excretion**	>24 h	2^[^ ^29^ ^,^ ^32^ ^]^	36	-0.2 (-0.5, 0.2)	μmol/min	0.32	0%
	≤24 h	4^[^ ^19^ ^,^ ^27^ ^,^ ^30^ ^]^	71	-0.1 (-0.3, 0.2)	μmol/min	0.51	2%
	All	6	107	-0.1 (-0.3, 0.1)	μmol/min	0.27	0%
**Kidney Weight: Body Weight Ratio**	>24 h	7^[^ ^21^ ^,^ ^23^ ^,^ ^24^ ^]^	103	-0.2 (-0.4, 0.0)	mg/g	0.08	0%
**Glomerular Volume**	>24 h	7^[^ ^21^ ^,^ ^23^ ^,^ ^24^ ^,^ ^29^ ^,^ ^32^ ^]^	123	-0.3 (-0.3, -0.2)	10^3^μm^3^	<0.00001	69%
**Glomerular Basement Membrane Thickness**	>24 h	3^[^ ^23^ ^,^ ^29^ ^]^	54	-3.2 (-19.2, 12.8)	nm	0.69	0%
**ECM fraction of Glomerular Cross Section**	>24 h	4^[^ ^23^ ^,^ ^24^ ^]^	69	-0.1 (-0.1, -0.1)	10^–2^	<0.00001	25%
**Plasma glucose**	>24 h	7^[^ ^21^ ^,^ ^26^ ^,^ ^28^ ^,^ ^29^ ^,^ ^32^ ^]^	107	-1.1 (-3.0, 0.7)	mmol/L	0.24	68%
	≤24 h	5^[^ ^18^ ^,^ ^19^ ^,^ ^27^ ^,^ ^30^ ^]^	85	-0.1 (-2.4, 2.2)	mmol/L	0.94	76%
	All	12	192	-0.7 (-2.0, 0.6)	mmol/L	0.29	69%
**Hemoglobin A1c**	>24 h	3^[^ ^21^ ^]^	35	-1.6 (-2.7, -0.5)	%	0.005	36%
**Mean Arterial Pressure**	>24 h	3^[^ ^21^ ^]^	35	2.8 (-2.1, 7.8)	mmHg	0.26	0%
	≤24 h	6^[^ ^18^ ^–^ ^20^ ^,^ ^27^ ^,^ ^30^ ^]^	103	-1.7 (-6.9, 3.4)	mmHg	0.51	4%
	All	9	138	0.6 (-3.0, 4.1	mmHg	0.76	0%

Positive mean difference indicates higher numbers in the C-peptide group. ECM = extracellular matrix. Unique Exp. = Number of unique experiments. I^2^ = heterogeneity (see methods). N = total number of animals.

Urine albumin excretion was reported in four unique experiments (n = 69 total animals from four publications) and urine protein excretion reported in three experiments (n = 48 total animals from three publications). Overall, there was no effect of C-peptide on albumin excretion (pooled mean difference -0.18 mg/day; 95% CI -0.57 to 0.21) but there was a significant reduction in urine protein excretion (pooled mean difference -186.25 mg/day; 95% CI -371.98 to -0.51) (Table 5). However, in studies <24 hours, both albuminuria and proteinuria were reduced by C-peptide. There was a significant increase in urine sodium excretion with C-peptide (pooled mean difference 0.21 μmol/min; 95% CI 0.0 to 0.42) but this effect was only seen in short-term studies lasting <24 hours (pooled mean difference 0.34 μmol/min; 95% CI 0.11to 0.57) (Table 5). There was no significant effect of C-peptide on urine potassium excretion (Table 5).

Glomerular volume and extra-cellular fraction of glomerular cross section were reported in seven (n = 123 total animals from 5 publication) and four (n = 69 total animals from 2 publications) unique experiments, respectively. C-peptide was associated with a significant reduction in glomerular volume (pooled mean difference -0.26 10^3^μm ^3^; 95% CI -0.34 to -0.18) and extra-cellular fraction of glomerular cross-section (pooled mean difference -0.08 x 10^–2^; 95% CI -0.10 to -0.07) (Table 5). Samnegard *et al*. [29] reported a reduction in mesangial matrix volume with C-peptide in diabetic animals, but this result was not pooled with the above due to different units. Glomerular basement membrane thickness was not affected by C-peptide (Table 5).

Notably there was no difference in mean arterial pressure or plasma glucose between animals receiving C-peptide or control (Table 5). However, C-peptide was associated with a significant reduction in HbA1c (pooled mean difference -1.56%; 95% CI -2.66 to -0.46) (Table 5). The data from Huang et al. [31] could not be included in the pooled analyses as they only reported percent change without raw data.

### C-Peptide in Non-diabetic Animals

**Fig 4:**
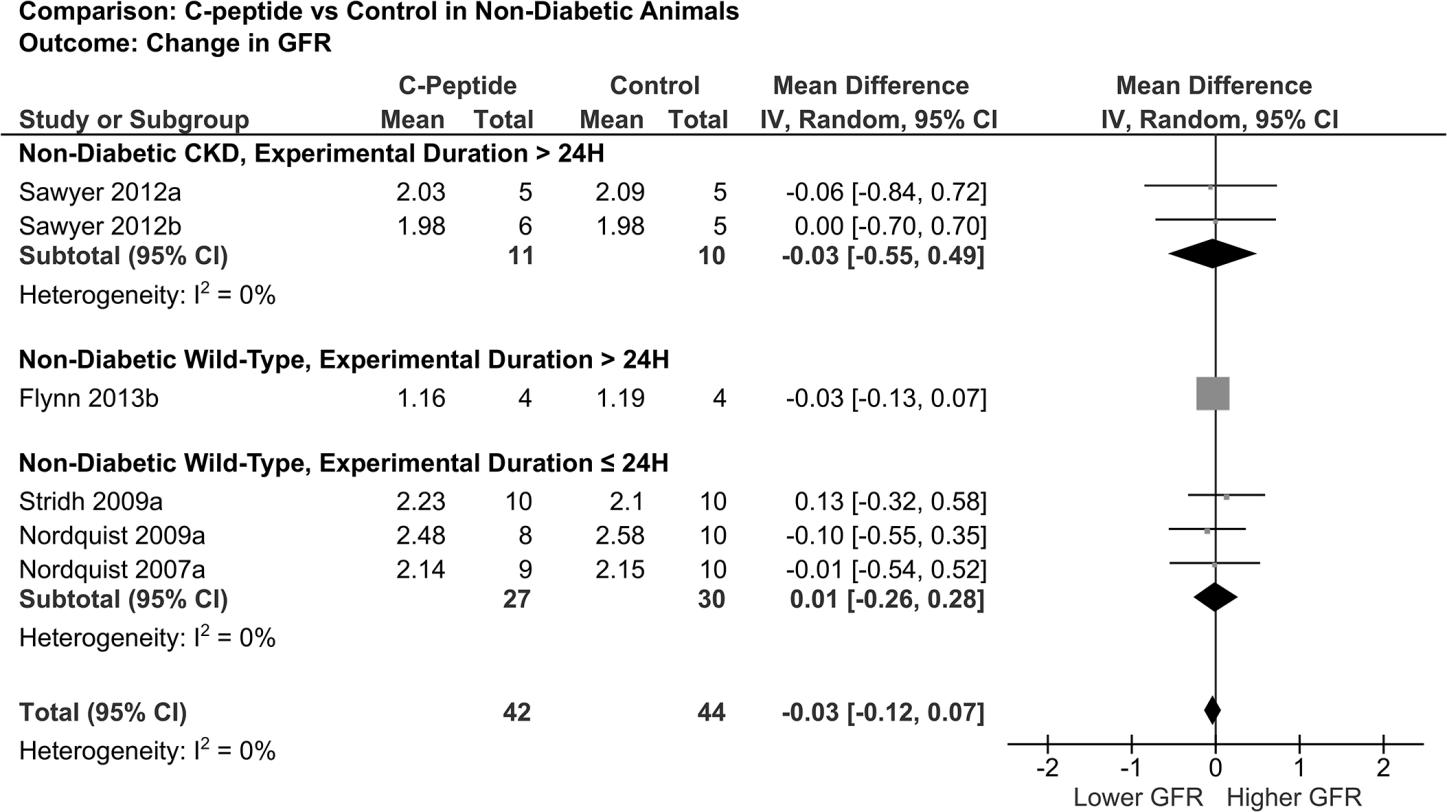
Forest plot showing pooled mean difference in GFR in non-diabetic animal studies. Sawyer 2012a = 2 weeks high-salt; Sawyer 2012b = 4 weeks high-salt. The box size reflects relative weight.

Only one study examined the renal effects of C-peptide in a model of non-diabetic CKD, the SS/jr rat [15]. Data involving C-peptide administration to non-diabetic wild-type animals was extracted from references [20–22,25,27]. GFR was not affected by C-peptide in SS/jr or wild-type animals as shown in Fig 4. Nakamoto *et al*. [22] did not observe a change in relative sieving coefficients with C-peptide compared to control in wild-type animals. Additionally, filtration fraction was unaffected by C-peptide in wild-type animals [20]. C-peptide did not affect renal plasma flow or renal vascular resistance (Table 6).

**Table 6:** Outcome summary of pooled mean differences in non-diabetic animal studies.

Outcome	Model & Duration	Unique Exp. [ref.]	N	Mean Difference (95% CI)	Units	P	I^2^
**Renal Plasma Flow**	WT, >24 h	1^[^ ^21^ ^]^	8	0.6 (-1.4, 2.6)	mL/min	0.56	
	WT, ≤24 h	1^[^ ^20^ ^]^	18	0.0 (-2.8, 2.8)	mL/min	1.00	
	All	2	26	0.4 (-1.2, 2.0)	mL/min	0.64	0%
**Renal Vascular Resistance**	WT, >24 h	1^[^ ^21^ ^]^	8	-0.2 (-0.9, 0.5)	mmHg/mL/min	0.54	
	WT, ≤24 h	1^[^ ^20^ ^]^	18	-1.1 (-4.2, 2.0)	mmHg/mL/min	0.49	
	All	2	26	-0.3 (-1.0, 0.4)	mmHg/mL/min	0.45	0%
**Urine Albumin Excretion**	NDCKD, >24 h	2^[^ ^15^ ^]^	21	-47.5 (-89.8, -5.1)	mg/day	0.03	0%
**Urine Protein Excretion**	NDCKD, >24 h	2^[^ ^15^ ^]^	21	-88.9 (-190.1, 12.4)	mg/day	0.09	48%
**Urine Na Excretion**	WT ≤24 h	1^[^ ^27^ ^]^	19	0.3 (-0.0,0.7)	μmol/min	0.07	
**Urine K Excretion**	WT ≤24 h	1^[^ ^27^ ^]^	19	0.8 (0.4, 1.1)	μmol/min	<0.0001	
**Kidney Weight: Body Weight Ratio**	NDCKD, >24 h	2^[^ ^15^ ^]^	21	-0.3 (-0.7, 0.1)	mg/g	0.14	0%
	WT, >24 h	1^[^ ^21^ ^]^	8	-0.7 (-1.1, -0.3)	mg/g	0.0004	
**Plasma glucose**	NDCKD, >24 h	2^[^ ^15^ ^]^	21	-0.1 (-0.8, 0.7)	mmol/L	0.81	0%
	WT, ≤24 h	1^[^ ^27^ ^]^	19	-0.4 (-0.8, 0.0)	mmol/L	0.07	
**Hemoglobin A1c**	WT, >24 h	1^[^ ^21^ ^]^	8	0.2 (-0.1, 0.4)	%	0.14	
**Mean Arterial Pressure**	NDCKD, >24 h	2^[^ ^15^ ^]^	21	-10.0 (-33.8, 13.9)	mmHg	0.41	0%
	WT, >24 h	1^[^ ^21^ ^]^	8	1.0 (-10.1, 12.1)	mmHg	0.86	
	WT, ≤24 h	2^[^ ^20^ ^,^ ^27^ ^]^	37	-1.3 (-6.4, 3.7)	mmHg	0.61	0%
	All (WT only)	3	45	-0.9 (-5.5, 3.7)	mmHg	0.69	0%

Positive mean difference indicates higher numbers in the C-peptide group. WT = wild-type animals; NDCKD = non-diabetic CKD. Unique Exp. = Number of unique experiments. I^2^ = heterogeneity (see methods). N = total number of animals.

C-peptide reduced both albuminuria and proteinuria in SS/jr rats pre-treated with 2 weeks of high salt (p<0.05), but did not affect either endpoint in animals pre-treated with 4 weeks of high salt, which is a more advanced disease state [15]. Pooled mean differences of both time-points as per our pre-specified protocol showed a statistically significant reduction in albuminuria (pooled mean difference -47.46 mg/day; 95% CI -89.82 to -5.1) but not proteinuria (p = 0.09) (Table 6). Urine sodium excretion was not affected by C-peptide in wild type animals, but urine potassium excretion was increased in one short term study (mean difference 0.76 μmol/min; 95% CI 0.38 to 1.14) (Table 6).

Kidney weight to body weight ratio was reported in three unique experiments. Two were in SS/jr rats and one in wild-type animals. C-peptide did not significantly affect renal size in the SS/jr rats pre-treated with 2 or 4 weeks of high salt, but did reduce the kidney weight to body weight ratio in one small experiment in wild-type animals (Table 6). Glomerular volume, GBM thickness, and extra-cellular fraction of glomerular cross section were not measured in non-diabetic animals. In non-diabetic animals, there was no effect of C-peptide on plasma glucose, HbA1c or blood pressure (Table 6). HbA1c data from Nakamoto *et al*. [22] were not included in our analysis due to unclear timing of its measurement.

### C-Peptide for Acute Kidney Injury in Animals

Two studies examined the effect of C-peptide on acute kidney injury in the setting of shock. In 2011, Chima *et al*. [16] examined the effect of intra-arterial C-peptide (dose: 280 nmol/kg/hr x 3hrs) following hemorrhagic shock in Wistar rats. Compared to vehicle control, animals receiving C-peptide had higher mean arterial pressure and lower plasma creatinine during and after resuscitation. In 2007, the same investigators reported that C-peptide improved survival in mice subjected to endotoxic shock compared to control, although no renal endpoints were examined [17].

## Discussion

There is very limited evidence at present supporting C-peptide therapy for patients with diabetic nephropathy, and no direct evidence to support its use in patients with other forms of CKD. Only four small human studies exist (n = 74 patients) that compare C-peptide to control and subsequently examine renal outcomes in humans, and all patients in these studies were type 1 diabetics with either normal GFR or glomerular hyperfiltration, with or without albuminuria. There are no human studies that examine the effect of C-peptide on renal function in more advanced CKD.

Our pooled analysis of post-intervention GFR revealed no difference between control and C-peptide; however, two parallel group studies reported a statistically significant decrease in glomerular hyperfiltration in the presence of C-peptide by comparing the change from baseline within each study group [6,13]. One possible explanation for this discrepancy is the small study sizes, which increases within-group variation and makes the detection of small differences difficult. A subsequent cross-over study by the same authors reported no difference in GFR during the first study period, although the baseline GFR at the start of this study was more varied (77–144 mL/min/1.73 m^2^), suggesting that C-peptide may have less effect when GFR is normal [5]. Additionally, discrepancies between the individual studies may be accounted for by unclear randomization in one study [13], different doses and duration of C-peptide therapy, and varying degrees of underlying renal disease in the study populations. Thus, the available data provide weak evidence that C-peptide may reduce glomerular hyperfiltration as well as microalbuminuria in type 1 diabetics with incipient nephropathy; however, this needs to be further characterized in subsequent larger randomized, placebo controlled trials. A reduction in glomerular hyperfiltration may be beneficial for these patients as a means to reduce glomerular hypertension, which is thought to contribute to tubulointerstitial fibrosis, mesangial expansion, glomerular sclerosis, and ultimately to the progression of renal disease towards end stage [33,34].

The animal literature has a larger body of data which supports the use of C-peptide for reduction of both glomerular hyperfiltration and proteinuria in diabetic models, but no evidence that C-peptide affects GFR in non-diabetic CKD. In a model of hypertensive nephrosclerosis, C-peptide reduced proteinuria in animals with mild/moderate disease, but had no effect on animals with more severe and perhaps irreversible disease. Reduction in other endpoints such as glomerular volume and mesangial matrix expansion lend further support to a therapeutic role for C-peptide in experimental diabetes, but these endpoints may not be as important as proteinuria and rate of GFR decline.

Plasma glucose data was highly heterogenous in both human and animals studies. We chose to ignore the presence or absence of concurrent insulin therapy when pooling diabetic animal data together, and this likely contributed to heterogeneity of these results. It has not been definitively established whether or not C-peptide affects blood sugar, nor whether any potential therapeutic mechanism for C-peptide is contingent upon the presence of insulin. Johansson *et al*. [6] reported a modest improvement in glycemic control with C-peptide, but this was not corroborated in a subsequent study by the same group [5]. In the present study, the decision to pool data in this manner was made to better reflect the diabetic patient population that we wish to treat, since there is variation in the degree of glycemic control among these patients.

In diabetic animals HbA1c was reduced in the presence of C-peptide, despite no change in glycemic control. Differences in experimental protocol, patient selection, or analytical methodology may account for these discrepancies, but also suggests that C-peptide may contribute in some way to glycemic control.

To our knowledge, this is the first systematic review looking at the potential role for C-peptide in kidney disease. Our sensitive search terms and broad inclusion criteria put this review at low risk for missing potentially important studies. However, limitations of our review include the small number and small size of the included human studies, the wide variation in individual study protocols, and the lack of human studies involving more advanced stages of CKD. That we did not include a pooled change from baseline analysis in our protocol could be seen as a limitation, but in order to perform this calculation we would have had to impute a standard deviation of the change, since this was not presented in the individual studies, and this may have introduced error into the results [12]. Several narrative reviews have been written on the physiological role of C-peptide and its effects on the kidney, peripheral nerves, vasculature, and inflammation, as well as its impact on intracellular signal transduction pathways in a variety of pathophysiological disease states [3,4,35–37]. Interestingly, the role of C-peptide in the setting of type 2 diabetes has not been well characterized. Higher levels of C-peptide in these patients have been associated with both metabolic syndrome [38], and with reduced risk of microvascular complications [39], and further research into the function of C-peptide in this context is needed.

The optimal dose of C-peptide for human therapy is not known and addressing this issue was not part of this review. In our analysis we pooled different doses together despite wide variation among studies. Regarding the C-peptide molecule itself, rat and human C-peptide share approximately 70% identity between the 31 amino acid sequences. Whether the interspecies sequence differences result in different physiological outcomes was not part of this review since we anticipate that human trials will continue to be done with human C-peptide.

One potential limiting factor in conducting larger human trials has been the short half-life of C-peptide. The human trials in this review either used a pump for continuous drug delivery or had multiple daily dosing. The former is effective but not practical for many patients, and the latter does not provide continuous drug exposure due to its short half-life of approximately 30–40 minutes in humans [40] and 20 minutes in rats [23]. The design of new fusion proteins or chemically modified C-peptide molecules that artificially lengthen the half-life, while preserving the physiological effects of the molecule, will be of benefit in future trials. Such agents are already in development [23,41].

From a mechanistic perspective, Nordquist et al have reported that C-peptide inhibits the activity of the renal Na+-K+-ATPase which accounts for the increase in urine sodium and reduction in glomerular hyperfiltration by reducing tubular Na reabsorption [20]. Achieving a better understanding of the underlying molecular effects of C-peptide is an area of active research.

Overall, the data support further study with larger double-blind, randomized, placebo-controlled trials to determine more definitively how C-peptide may affect renal function in patients with CKD. The patient population studied so far has been relatively young people with type 1 diabetes with minimal, if any, diabetic nephropathy. It would be useful for future trials to include patients with more advanced CKD to see if C-peptide could be a useful therapeutic tool in this population. For example, Flynn *et al*. [21] showed that even after a period of untreated diabetic nephropathy, C-peptide was able to reduce proteinuria in rats, which suggests that this therapy may be appropriate for patients with more advanced diabetic nephropathy. It would also be worthwhile to include patients with other non-diabetic forms of proteinuric CKD, such as hypertensive nephrosclerosis, to ascertain if these patients would also benefit from C-peptide. These hypothetical studies should ideally be of sufficient duration to be able to capture a change in the speed of progression to end stage or need for renal replacement therapy.

In addition to CKD, the beneficial effects of C-peptide in animal models of distributive and hypovolemic shock highlight another potential therapeutic role for C-peptide in the acute critical care setting. There have been several reports looking at the anti-inflammatory role of C-peptide showing improved end-organ function in the acute setting in the lung, heart, and kidney [16,17,42,43]. Whether this effect will translate to improved outcomes for patients with shock in an intensive care setting has not yet been investigated, but a small pilot study comparing placebo to C-peptide in an ICU may yield interesting results.

It is our hope that this review on the therapeutic use of C-peptide for patients with renal impairment of any etiology will guide further hypothesis generation and subsequent studies towards the goal of slowing progression of renal disease and reducing the burden of renal replacement therapy.

## Supporting Information

S1 File Full-text excluded articles with reasons for exclusion (N = 20). (DOCX)

S2 File PRISMA Checklist. (PDF)

S1 Table Risk of bias in animal studies. Yes = lower risk of bias.
(DOCX)
