# Supplementary material for: C-peptide as a Therapy for Kidney Disease: A Systematic Review and Meta-Analysis
Source: PLoS One. 2015 May 20;10(5):e0127439. doi: 10.1371/journal.pone.0127439 (PMC4439165; doi:10.1371/journal.pone.0127439)
Supplement: S1 File — (DOCX) [file pone.0127439.s001.docx]

**S1 File. Full-text excluded articles with reasons for exclusion (N=20).**

Full-text articles excluded due to non-renal outcomes (N=9)

1. Lim Y-, Bhatt MP, Kwon M-, Park D, Lee S, Choe J, et al. Prevention of VEGF-mediated microvascular permeability by C-peptide in diabetic mice. Cardiovasc Res. 2014;101:155-164.
2. Bhatt MP, Lim Y-, Hwang J, Na S, Kim Y-, Ha K-. C-peptide prevents hyperglycemia-induced endothelial apoptosis through inhibition of reactive oxygen species-mediated transglutaminase 2 activation. Diabetes. 2013;62:243-253.
3. Foyt H, Daniels M, Milad M, Wahren J. Pharmacokinetics, safety, and tolerability of a long-acting C-peptide (CBX129801) in patients with type 1 diabetes. Diabetologia. 2012;55:S455.
4. Callaway J, Martensson A, Mazzoni M, Barrack S, Wahren J. Development of a long-acting C-peptide. Diabetes. 2011;60:A288.
5. Wasada T, Kuroki H, Arii H, Maruyama A, Katsumori K, Aoki K, et al. Hyperglycemia facilitates urinary excretion of C-peptide by increasing glomerular filtration rate in non-insulin-dependent diabetes mellitus. Metabolism: Clinical and Experimental. 1995;44:1194-1198.
6. Hoogwerf BJ, Goetz FC. Urinary fractional excretion (U(FE)) and metabolic clearance of infused synthetic human C-peptide (CP) in insulin dependent diabetic (IDD) subjects with normal and impaired renal function. Diabetes. 1982;31:No. 539.
7. Rubenstein AH, Steiner DF, Horwitz DL, Mako ME, Block MB, Starr JI, et al. Clinical significance of circulating proinsulin and C-peptide. Recent Prog Horm Res. 1976;33:435-475.
8. Stevens MJ, Zhang W, Li F, Sima AA. C-peptide corrects endoneurial blood flow but not oxidative stress in type 1 BB/Wor rats. Am J Physiol Endocrinol Metab. 2004;287:E497-505.
9. Cotter MA, Ekberg K, Wahren J, Cameron NE. Effects of proinsulin C-peptide in experimental diabetic neuropathy: vascular actions and modulation by nitric oxide synthase inhibition. Diabetes. 2003;52:1812-1817.

Full-text articles excluded due for being a conference abstract (N=6)

1. Maric C, Flynn ER, Chade AR. Treatment with C-peptide slows the progression of diabetic renal disease in the streptozotocin (STZ)-induced diabetic rat. FASEB Journal. 2011;25. Conference: Experimental Biology 2011. Washington, DC United States.
2. Nakamoto H, Yada T, Ogasawara Y, Kajiya F. Glomerular filtration at the early stage of diabetes. FASEB Journal. 2011;25. Conference: Experimental Biology 2011. Washington, DC United States.
3. Pihl L, Palm F, Nordquist L. Proinsulin C-peptide protects against renal ischemia-reperfusion injury in diabetic rats. FASEB Journal. 2011;25. Conference: Experimental Biology 2011. Washington, DC United States.
4. Zerbini G, Meschi F, Bonfanti R, Viscardi M, Rigamonti A, Frontino G, et al. Role of c-peptide in the pathogenesis of microvascular complications in type 1 diabetes with pediatric age onset. Pediatric Diabetes. 2011;12:46. Conference: 37th Annual Meeting of the International Society for Pediatric and Adolescent Diabetes. Miami Beach, FL United States.
5. Piraino G, Maltese G, LaMontagne T, Hake PW, Denenberg A, O'Connor M, et al. C-peptide exerts beneficial effects in kidney injury following hemorrhagic shock. Shock. 2010;33:44. Conference: 33rd Annual Conference on Shock. Portland, OR United States.
6. Regeur L, Binder C. The correlation between plasma C peptide and kidney function. Diabetologia. 1976;12:No. 223.

Full-text articles excluded for *in vitro* or *ex vivo* experiments or molecular outcomes only (N=5)

1. Li Y, Zhao M, Li B, Qi J. Dynamic localization and functional implications of C-peptide might for suppression of iNOS in high glucose-stimulated rat mesangial cells. Mol Cell Endocrinol. 2013;381:255-260.
2. Nordquist L, Shimada K, Ishii T, Furuya DT, Kamikawa A, Kimura K. Proinsulin C-peptide prevents type-1 diabetes-induced decrease of renal Na+-K+-ATPase alpha1-subunit in rats. Diabetes Metab Res. 2010;26:193-199.
3. Nordquist L, En YL, Sjoquist M, Patzak A, Persson AEG. Proinsulin C-peptide constricts glomerular afferent arterioles in diabetic mice. A potential renoprotective mechanism. American Journal of Physiology - Regulatory Integrative and Comparative Physiology. 2008;294:R835-R841.
4. Maestroni A, Ruggieri D, Dell'Antonio G, Luzi L, Zerbini G. C-peptide increases the expression of vasopressin-activated calcium-mobilizing receptor gene through a G protein-dependent pathway. European Journal of Endocrinology. 2005;152:135-141.
5. Lindstrom K, Johansson C, Johnsson E, Haraldsson B. Acute effects of C-peptide on the microvasculature of isolated perfused skeletal muscles and kidneys in rat. Acta Physiol Scand. 1996;156:19-25.
