# Supplementary material for: C-peptide as a Therapy for Kidney Disease: A Systematic Review and Meta-Analysis
Source: PLoS One. 2015 May 20;10(5):e0127439. doi: 10.1371/journal.pone.0127439 (PMC4439165; doi:10.1371/journal.pone.0127439)
Supplement: S1 Table — Yes = lower risk of bias. (DOCX) [file pone.0127439.s003.docx]

**S1 Table. Risk of bias in animal studies.**

| **Reference** | **Adequate sequence generation?** | **Allocation Concealment?** | **Blinding?** | **Incomplete Outcome Data Addressed?** | **Free of Selective Reporting?** | **Free of other sources of bias?** |
| --- | --- | --- | --- | --- | --- | --- |
| **Flynn *et al.* [21]** | Yes | Unclear | Unclear | Yes | Yes | Yes |
| **Nakamoto *et al.* [22]** | Unclear | Unclear | Unclear | Yes | Yes | Yes |
| **Sawyer *et al.* [15]** | Yes | Unclear | Yes | Yes | Yes | Yes |
| **Yang *et al.* [23]** | Unclear | Unclear | Unclear | Yes | Yes | Yes |
| **Chima *et al.* [16]** | Unclear | Unclear | Unclear | Yes | Yes | Yes |
| **Sun *et al.* [24]** | Unclear | Unclear | Unclear | Yes | Yes | Yes |
| **Stridh *et al.* [25]** | Yes | Unclear | Unclear | Yes | Yes | Yes |
| **Nordquist *et al.* [20]** | Unclear | Unclear | Unclear | Yes | Yes | Yes |
| **Kamikawa *et al.* [26]** | Yes | Unclear | Unclear | Yes | Yes | Yes |
| **Nordquist *et al.* [27]** | Unclear | Unclear | Unclear | Yes | Yes | Yes |
| **Vish *et al.* [17]** | Yes | Unclear | Unclear | Yes | Yes | Yes |
| **Maezawa *et al.* [18]** | Unclear | Unclear | Unclear | Yes | Yes | Yes |
| **Rebsomen *et al.* [28]** | Unclear | Unclear | Unclear | Yes | Yes | Yes |
| **Samnegard *et al.* [29]** | Unclear | Unclear | Unclear | Yes | Yes | Yes |
| **Samnegard *et al.* [30]** | Unclear | Unclear | Unclear | Yes | Yes | Yes |
| **Huang *et al.* [31]** | Unclear | Unclear | Unclear | Yes | Yes | Yes |
| **Samnegard *et al.* [32]** | Unclear | Unclear | Unclear | Yes | Yes | Yes |
| **Sjöquist *et al.* [19]** | Unclear | Unclear | Unclear | Yes | Yes | Yes |

Yes = lower risk of bias
